# Genome-Wide Analysis and Expression Profiles of AhCOLs Family in Peanut (*Arachis hypogaea* L.)

**DOI:** 10.3390/ijms26073404

**Published:** 2025-04-05

**Authors:** Wei Wang, Xiaoyu Liu, Che Liu, Xiaoqin Liu

**Affiliations:** 1 Peking University Institute of Advanced Agricultural Sciences, Shandong Laboratory of Advanced Agriculture Sciences at Weifang, Weifang 261325, China; wei.wang@pku-iaas.edu.cn (W.W.); liuxiaoyu202204@163.com (X.L.); che.liu@pku-iaas.edu.cn (C.L.); 2 College of Life Sciences, Shandong Agricultural University, Taian 271018, China

**Keywords:** *Arachis hypogaea*, *AhCOL*, gene family, flowering

## Abstract

The CONSTANS-like (COL) gene family plays critical roles in plant growth, development, stress responses, and light signal transduction. However, its functions in peanut (Arachis hypogaea) remain poorly understood. In this study, we identified 18 AhCOL genes in the peanut genome, all localized in the nucleus. Phylogenetic analysis classified these genes into three subfamilies, with Group I containing eight members and Groups II and III each comprising five. Conserved domain analysis revealed that all AhCOL proteins possess at least one B-box and one CCT domain. Most of the AhCOL members in peanuts contain a large number of ABA and MeJA elements. Additionally, some members have low-temperature response elements, anaerobic induction, circadian control, and defense stress elements. Expression profiling indicated that most *AhCOL* genes are abundantly expressed in leaves, flowers, and fruit needles. Notably, genes such as *AhCOL4*, *AhCOL8*, *AhCOL13*, and *AhCOL14* were upregulated under light induction and mechanical stress, highlighting their involvement in pod development. AhCOL1 interacts with AhNF-YC1, while AhCOL3 interacts with both AhNF-YC1 and AhCOP1 proteins. This study identifies key AhCOL genes implicated in light and mechanical stress responses, offering insights into their potential roles in peanut flowering and abiotic stress tolerance.

## 1. Introduction

Peanuts are a globally significant legume crop valued for their nutritional and bioactive compounds, including premium oils, proteins, resveratrol, isoflavones, and phytic acids [1]. It ranks as the third most important source of plant-derived protein [2]. Cultivated peanut (*Arachis hypogaea* L.), an allotetraploid species (AABB, 2n = 4x = 40), originated from hybridization and polyploid differentiation of *Arachis duranensis* (AA genome) and *Arachis ipaensis* (BB genome) [3]. Peanuts are one of the most important economic crops in China, and their yield, quality, and resistance have attracted much attention. The growth period directly determined by the flowering time of peanuts is closely related to the yield of peanuts, so it is necessary to understand how the flowering of important crops such as peanuts is regulated.

Peanuts are a crop that blossoms on the ground and bears pods underground. Flowering time is one of the important indexes to evaluate early maturing peanut varieties, and it also has an important impact on yield [4]. Production is affected by its own habits and external environmental conditions. In order to obtain high and stable yield of peanut, it is necessary to study the flowering habits of peanut and master its flowering rules [5]. A QTL related to the flowering of the main stem was located on LG06 using *A. duranensis* and *A. cardenasii* as parents, which explained 44% of the phenotypic variation [6]. In peanuts, 390 natural populations of peanut cultivars were used as materials, 259 SNP loci significantly associated with flowering time were obtained through genome-wide association analysis, and seven candidate genes associated with flowering time were predicted in the candidate intervals of repeated loci encoding R2R3-MYB, chlorophyll a-b binding protein, bHLH, WRKY, and FAR1 [7]. Previous studies have reported that PEBPs are involved in the regulation of flowering time and various developmental processes. Multiple PEBP genes have different expression patterns in early-flowering and late-flowering genotypes [8]. However, there is no report on the cloning of functional genes for flowering habits in peanuts. Therefore, it is necessary to systematically study the key genes related to flowering habits of peanuts and lay a foundation for subsequent analysis of a flowering regulation genetic mechanism of peanuts and the early breed of the peanut. The yield of peanuts can be improved by studying the molecular pattern of flowering genes and increasing the effective flowering time.

In higher plants, the transition from vegetative to reproductive growth, termed floral transition, is a critical phase for crop development. This transition, governed by flowering time [9], directly impacts crop yield. Flowering is regulated through the interaction of endogenous and environmental cues [10]. CONSTANS (CO), a key regulator in the photoperiod pathway, plays a central role in the flowering regulatory network. Flowering is orchestrated by complex networks involving pathways such as photoperiod, gibberellin, vernalization, aging, and autonomy [11,12,13]. Among these, the photoperiod pathway is particularly prominent, enabling plants to sense day length and trigger downstream molecular responses [14]. CONSTANS-like (*COL*) genes, belonging to the zinc finger transcription factor family, play crucial roles in plant growth and development [15]. COL proteins are characterized by conserved domains, including one or two B-box (BBX) domains and a CCT (CONSTANS, CO-like, TIMING OF CAB1) domain [16]. BBX domains, classified as B-Box1 or B-Box2 based on their structural features, are involved in protein–protein interactions [17]. The CCT domain regulates transcription and nuclear transport [17,18]. COL proteins are categorized into three classes based on their domain composition: Classes I and II possess two BBX domains and a CCT domain, while Class III contains one BBX and one CCT domain. Additionally, some COL proteins feature a valine–proline (VP) motif at their C-terminus [15,16]. In Arabidopsis, Classes I, II, and III comprise 6, 7, and 4 members, respectively.

Despite extensive research on COL genes in other plants, studies on *AhCOL* genes in peanuts remain limited. To date, there has been no comprehensive or systematic analysis of the AhCOL gene family in peanuts, restricting our understanding of its roles in development. This study employs bioinformatics to analyze *AhCOL* genes, providing a detailed characterization of their expression patterns across tissues and under light and mechanical stress. The findings lay a theoretical foundation for exploring the roles of *AhCOL* genes in flowering and abiotic stress responses in peanuts.

## 2. Results

### 2.1. Physical and Chemical Properties and Chromosomal Localization of the AhCOL Gene Family

**Figure 1:**
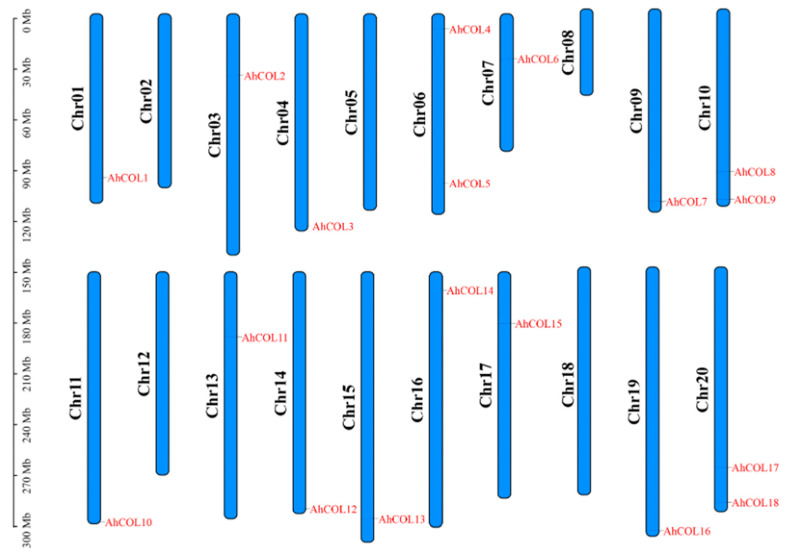
The location of the AhCOL gene family members in peanuts. Chromosomes 1–20 are shown in long blue bars. AhCOL genes are shown on the left of each chromosome. Gene positions and the size of each chromosome can be estimated using the scale on the left of the figure.

*AhCOL* gene family members were identified by HMMER software (version 3.3.2), and the results showed that a total of 18 *AhCOL* gene family members were identified in the peanut genome and named based on their homology with *Arabidopsis thaliana* genes and chromosomal locations. The distribution of the *AhCOL* gene family across peanut chromosomes was relatively uniform, with Chr02, Chr05, Chr08, Chr12, and Chr18 lacking *AhCOL* genes. Other chromosomes contained one to two genes (Figure 1). The physical and chemical properties of the *AhCOL* gene family were analyzed, revealing that amino acid lengths ranged from 327 to 434, with molecular weights spanning 35.69–48.58 kDa (average: 43.04 kDa). The theoretical isoelectric points ranged from 4.82 to 6.96 (mean: 5.83). Subcellular localization analysis predicted all *AhCOL* genes to localize to the nucleus (Table 1).

### 2.2. Phylogenetic Analysis of AhCOL Gene Family in Peanut

**Figure 2:**
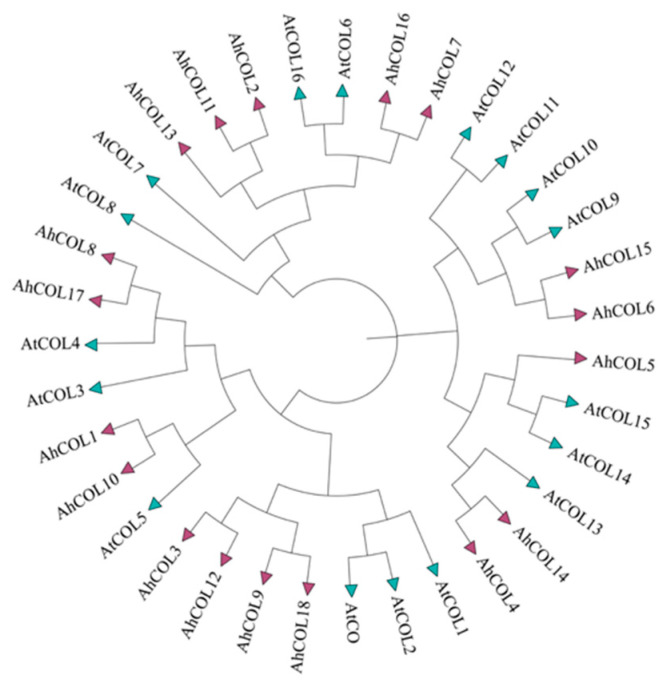
Phylogenetic tree of 18 *AhCOL* gene family in peanut. Phylogenetic tree was constructed using the neighbor-joining method by MEGA11.

Phylogenetic analysis was performed using AhCOL protein sequences from peanuts and *A. thaliana*. The analysis revealed that the AhCOL family in peanuts is divided into three subfamilies (Figure 2). Subfamily I included the largest number of genes with a total of 8 members. *AhCOL1*, *AhCOL3*, *AhCOL8*, *AhCOL9*, *AhCOL10*, *AhCOL12*, *AhCOL17*, and *AhCOL18* genes. However, the numbers of AhCOL family gene members in groups II and III were lower, and they all contained five gene family members.

### 2.3. Genetic Structure Analysis of AhCOL Genes in Peanut

**Figure 3:**
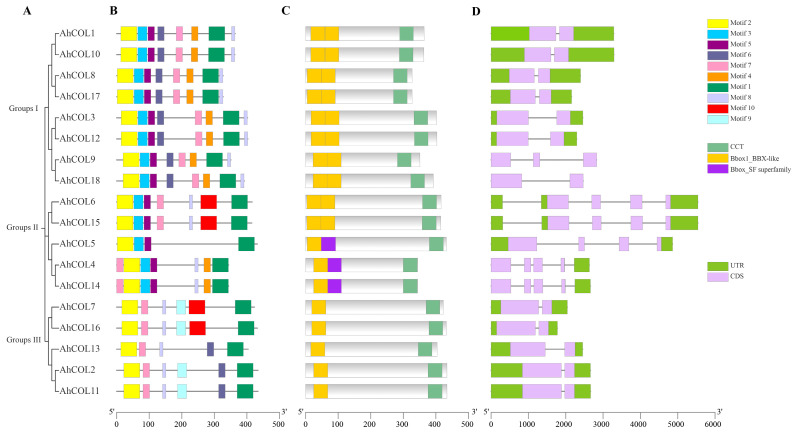
Gene structure of *AhCOL* genes in peanuts. (**A**), Phylogenetic tree of *AhCOL* gene family in peanut. (**B**), Distribution of 10 conserved motifs in *AhCOL* gene analyzed by MEME. The motifs were labeled on the right top. (**C**). Analysis of conserved domains of *AhCOL* genes in peanuts. The conserved domains were labeled on the right top. (**D**), *AhCOL* genetic structure analysis. The yellow rectangle represents the exon, the light green rectangle represents the UTR, and the gray line linking the two exons represents the intron.

The conserved domains and motifs of the AhCOL proteins were identified, with all members containing at least one B-box and CCT domain. MEME analysis highlighted conserved motifs, with motif (B-box: LTDMDREARVLRYREKKKTRKFEKKIRYESRKAYAETRPRIKGRFAKRTD) and motif 2 (CCT: PRACDSCKSARAVVYCRADSAFLCSACDSKVHSANQLASRHERVWLCEAC) being core motifs present in all AhCOL proteins (Figure 3B and Figure S1). Although the motif distribution is not stable, the motifs in the same group are highly conserved. For example, both groups I and III each contain motif1, motif2, motif3, motif5, and motif8. Group I contains motif6 in addition to the motifs described above. In addition to AhCOL13, motif9 is unique in Group II. This also indicates that AhCOL13 in peanuts may have unique biological functions (Figure 3C). Analysis of the gene structure of AhCol in peanuts showed that Group I and II members have a relatively simple genetic structure. Group I members have two to three exons; Group II members have one to two exons. Members of Group III had three to four exons (Figure 3D). Combined with the phylogenetic tree, the exon/intron pattern distributions of the same subgroup were more similar (Figure 3A). These findings provide further evidence for classification verification according to phylogenetic analysis.

### 2.4. Collinearity Analysis of AhCOL Gene Family in Peanut

**Figure 4:**
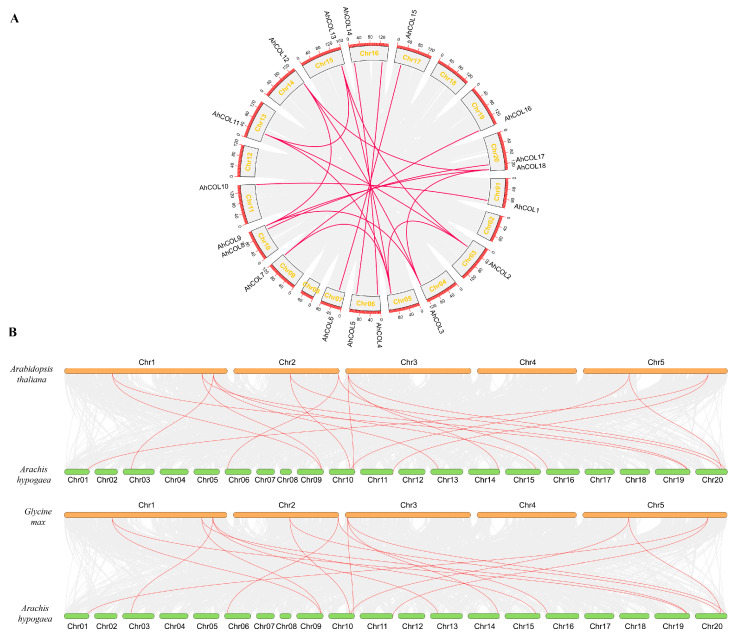
Synteny analysis of the *AhCOL* gene family in peanut. (**A**) Schematic representation of the interchromosomal relationships between the AhCOL genes in the Peanut (*Arachis hypogaea* L.) genome. (**B**) Synteny analysis of the COL genes between peanut (*Arachis hypogaea* L.) and 2 representative species. Red lines highlight the colinear gene pair, while gray lines indicate the syntenic blocks.

**Figure 5:**
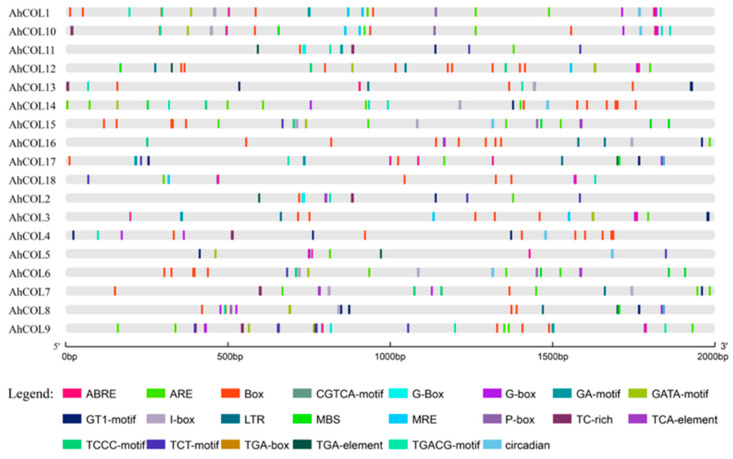
Analysis of *AhCOL* gene family promoter elements. Different *cis*-regulatory elements in the promoter are represented by square bars of various colors. The light responsive cis-elements mainly include the GATA-motif, G-Box, Box4, GT1, and circadian rhythm; Hormone responsive: TGACG-motif, P-box, ABRE, TCA-element, and TGA-element; Stress responsive: LTR, TC-rich repeats, MBS, and sARE; Growth regulation: CAT-box and GCN4 motif; Common cis-acting: CAAT-box; Core promoter: TATA-box.

Collinearity analysis was used to understand the evolutionary relationship of AhCOL gene family. Gene duplication is a primary mechanism for the emergence of new functional genes and a critical driving force in species evolution. In this study, the homology of *AhCOL* gene family members in peanut was found to be high. *AhCOL2*, *AhCOL3*, *AhCOL9*, *AhCOL11*, *AhCOL12*, *AhCOL12*, and *AhCOL18* contain three pairs of homologous genes. *AhCOL7* contains two pairs of homologous genes. The remaining members each have one pair of homologous genes (Figure 4). We also compared the synteny of *Arabidopsis thaliana*, soybean, and peanut genomes. The results revealed that most *AhCOL* genes in peanuts had only one homologous gene in *Arabidopsis* within the synteny relationship. Soybeans and peanuts exhibited higher homology, with a more complex synteny relationship. Most of the peanut *AhCOL* genes had multiple homologous genes in soybean (Figure 5).

Collinearity analysis of the *AhCOL* gene family between peanuts and other species. The gray lines represent collinearity between all members, and the collinearity pairs were connected with red lines. The species and chromosome numbers were indicated near each chromosome.

### 2.5. Cis-Element Analysis of AhCOL Gene Promoters in Peanut

The *cis*-regulatory elements of peanut *AhCOL* gene promoters were analyzed. The results showed that these elements mainly included light-responsive elements, hormone-responsive elements, growth and development-related elements, and low-temperature defense elements. The promoter regions of the *AhCOL* gene family contain the largest number of light-responsive elements (Figure S2). All *AhCOL* genes possess light-responsive elements, including Box 4 (ATTAAT), G-Box (CACGTG), and GT1 (GGTTAA). The promoters of *AhCOL2*, *AhCOL6*, *AhCOL7*, *AhCOL15*, and *AhCOL16* contain SA-response elements (“TCA-element”). All members contain ABA-responsive elements, except *AhCOL2*, *AhCOL6*, *AhCOL7*, *AhCOL11*, *AhCOL14*, *AhCOL15*, and *AhCOL16*. Similarly, all members contain MeJA-responsive elements, except *AhCOL3*, *AhCOL5*, *AhCOL6*, *AhCOL7*, *AhCOL12*, *AhCOL15*, and *AhCOL16*. Additionally, some members have low-temperature response elements, anaerobic induction, circadian control, and defense stress elements (Figure 5). These findings suggest their potential roles in responses to light signals, defense, stress, and developmental regulation.

### 2.6. Expression of AhCOL Genes in Different Tissues of Peanut

**Figure 6:**
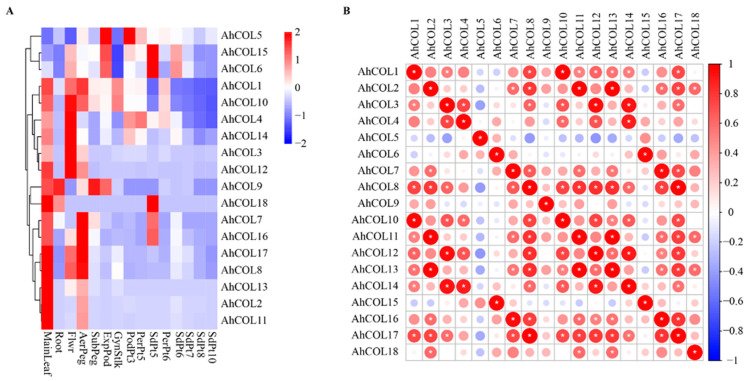
Expression profiles of AhCOL genes among 15 tissues. (**A**) Expression patterns of the AhCOL gene family in various tissues of peanut. Main leaf, main stem leaf; Root, roots of 10 days postemergence; Flwr, petals, keel, and hypanthium sepals; AerPeg, elongating aerial pegs; Subpeg, elongating subterranean pegs; ExpPod, Pattee 1 pod; GynStlk, Pattee 1 stalk of gynophore; PodPt3, Pattee 3 pod; PerPt5, Pattee 5 pericarp; SdPt5, Pattee 5 seed; PerPt6, Pattee 6 pericarp; SdPt6, Pattee 6 seed; SdPt7, Pattee 7 seed; SdPt8, Pattee 8 seed; SdPt10, Pattee 10 seed [19]. (**B**) Correlation analysis of AhCOL gene pairs among the 15 tissues. Asterisks represent significant differences (*p* < 0.05).

To determine the expression patterns of *AhCOL* family members in different tissues of peanuts, transcriptome analysis was performed on 15 tissues. The results revealed that most *AhCOL* genes were expressed in leaves, flowers, and fruit needles. Only two genes, *AhCOL9* and *AhCOL18*, were highly expressed in roots. *AhCOL5*, *AhCOL6*, *AhCOL9*, and *AhCOL15* were abundantly expressed during ExpPod development (Figure 6A). To investigate differences in the expression of duplicated genes, we analyzed the correlation of *AhCOL* segment-duplicated gene expression across tested tissues. The results indicated a significant correlation for most duplicated gene pairs. However, exceptions were observed, such as *AhCOL9* and *AhCOL7*, *AhCOL11* and *AhCOL8*, and *AhCOL22* and *AhCOL19*. Other gene pairs, such as *AhCOL5* and *AhCOL4*, and *AhCOL6* and *AhCOL3*, displayed significant differences (Student’s *t*-test, *p* < 0.05) (Figure 6B). Expression correlation was high across different tissues, but *AhCOL* gene family members exhibited lower correlation coefficients during flower and SdPt5 stages (Figure S3).

### 2.7. Expression of Peanut AhCOL Gene Family Members Under Light and Mechanical Pressure

**Figure 7:**
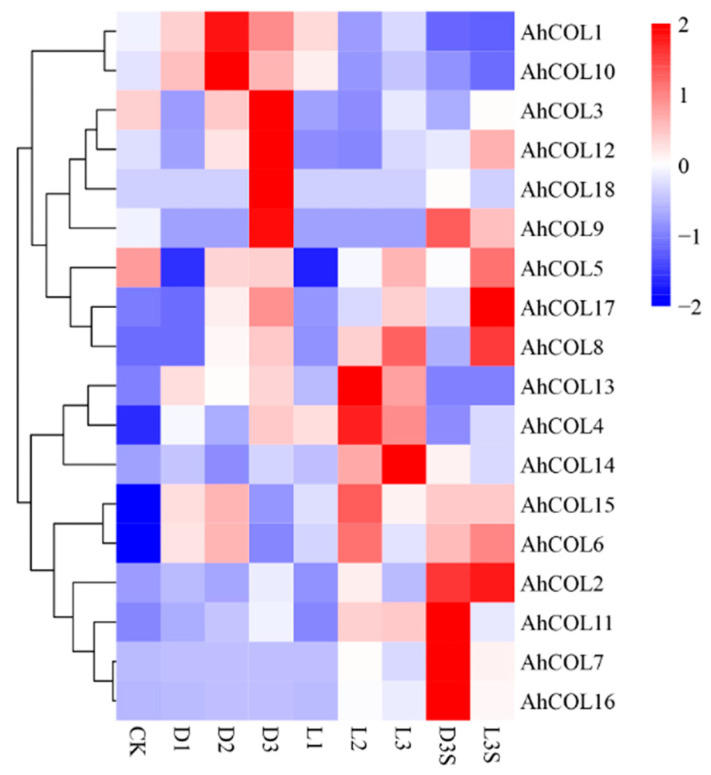
Expression of peanut *AhCOL* gene family members under light and mechanical stress.

To explore the expression patterns of *AhCOL* genes under light and mechanical pressure, the transcriptome of peanut pods treated with light and mechanical pressure was analyzed. The results revealed that *AhCOL1*, *AhCOL3*, *AhCOL10*, and *AhCOL12* were highly expressed in the dark. *AhCOL4*, *AhCOL8*, *AhCOL13*, *AhCOL14*, and other genes were induced in the absence of mechanical pressure, with more pronounced expression under light induction. Similarly, *AhCOL2*, *AhCOL7*, *AhCOL11*, and *AhCOL16* were highly expressed in the pericarp, but their expression decreased under light stress (Figure 7).

CK (dark and mechanical stress), the D (dark and lose mechanical stress) samples harvested after 10 (day 1), 34 (day 2), and 58 (day 3) h of treatment were named D1, D2, and D3, respectively. The L (light and lose mechanical stress) samples were harvested after 10 (day 1), 34 (day 2), and 58 (day 3) h of treatment and were named L1, L2, and L3. The pod shells of the sample DM, D1, D2, D3, L1, L2, L3, and the seed of sample D3 (named D3S) and L3 (named L3S). Two biological replicates were performed for each condition.

### 2.8. Subcellular Localization of AhCOL Proteins in Peanut

**Figure 8:**
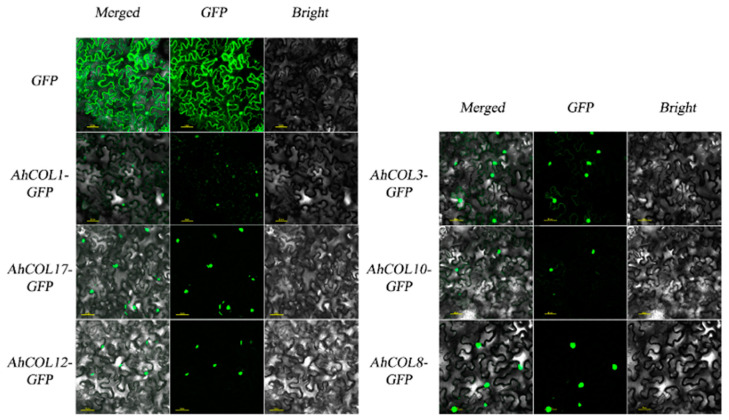
Subcellular localization of *AhCOL* genes in peanuts. The constructed fusion vector (35S: AhCOL-GFP) and control vector (35S: GFP) were transferred into tobacco by injection. GFP: GFP fluorescence; Bright: Bright field; Merged: Combined image of GFP. Scale bars are 50 μm in the images. Green color indicates GFP signal.

*AhCOL* proteins localization was performed by subcellular localization experiments. We cloned eight genes from peanut *AhCOL* gene family group 1. The sequences of six members *AhCOL1*, *AhCOL3*, *AhCOL8*, *AhCOL10*, *AhCOL12*, and *AhCOL17* were successfully obtained and cloned into the 1300 plant expression vector, generating constructs 1300-*AhCOL1*-GFP, 1300-*AhCOL3*-GFP, 1300-*AhCOL8*-GFP, 1300-*AhCOL10*-GFP, 1300-*AhCOL12*-GFP, and 1300-*AhCOL17*-GFP. These vectors were transiently expressed in 2–3 week-old tobacco leaves via *Agrobacterium*-mediated transformation. The results showed that AhCOL1, AhCOL3, AhCOL8, AhCOL10, AhCOL12, and AhCOL17 localized to the nucleus, as shown in Figure 8.

### 2.9. Peanut AhCOL Protein Interaction

**Figure 9:**
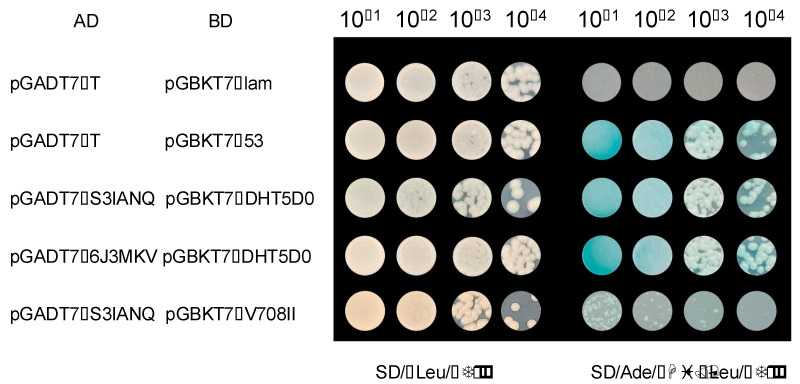
Interacting proteins with AhCOL in peanuts. In Y2H assay, the coding sequences of *AhCOL1*, and *AhCOL3* were ligated to activation domain vectors (AD, pGADT7-*AhCOL1*, and pGADT7-*AhCOL3*), and coding sequences of *NF*-*YC1* and *COP1* were fused to GAL4 DNA-binding domain vectors, (BD, pGBKT7-*COP1*, pGBKT7-*N*-*YC1*). Positive control: (Y2HGold [pGBKT7-53 + pGADT7-T]). Negative control: (Y2HGold [pGBKT7-Lam + pGADT7-T]). SD-Leu-Trp and SD-Ade-His-Leu-Trp medium were used of Y2H.

To further explore interactions involving peanut AhCOL proteins, we performed protein interaction network analysis on AhCOL1 and AhCOL3. The results showed that AhCOL1 protein interacted with AhGIGANTEA, AhNF-YC, and AhNF-YB proteins (Figure S4). AhCOL3 protein interacts with AhGIGANTEA, AhPeptidaseC19, AhPTPLA, and AhHAG702 proteins (Figure S5). The interacting proteins of AhCOL protein were verified by yeast two-hybrid. We constructed the AhCOL genes into the bait vector pGBKT7 using homologous recombination. Potential factors involved in the peanut flowering pathway were selected, and their full-length CDSs were cloned into the pGADT7 vector for yeast two-hybrid (Y2H) experiments. These predicted genes were tested for interactions with AhCOL1 and AhCOL3 using Y2H. The results revealed that AhCOL1 interacts with AhNF-YC1, while AhCOL3 interacts with both AhNF-YC1 and AhCOP1 proteins (Figure 9).

## 3. Discussion

The *AhCOL* gene is a pivotal component of the epigenetic regulatory network governing plant growth, development, and responses to abiotic stress. However, its role in peanuts remains unclear. In this study, 18 *AhCOL* genes were identified in the peanut genome by bioinformatics. There are some differences in the number of COL genes among different species: 17 in *Arabidopsis* [20], 16 in rice [21], 15 in *Petunia axillaris*, 18 in *Petunia inflata* [22], and 11 in foxtail millet (*Setaria italica*) [23]. Phylogenetic tree analysis showed that Col genes could be divided into three groups in the peanut group, which is similar to the grouping in rice and *Arabidopsis* [16]. Genes clustered in the same group in the phylogenetic tree showed the same motif and similar number of exons. For example, members in “Group II” have the same number of exons. *AhCOL2*, *AhCOL7*, *AhCOL11*, *AhCOL13*, and *AhCOL16* in “Group III” have the same conserved domains and the same number of exons, introns. Different groups of AhCOL members showed differences in exon–intron and motif distribution. These differences may be the reasons for their different biological functions. Collinearity analysis showed that peanuts had high homology with Arabidopsis and soybean. The results showed that the COL gene in peanuts was conservative in the evolutionary relationship.

Col transcription factors have been reported to be involved in the regulation of flowering time in the photoperiod signaling pathway. In addition, the Col gene family also plays an important role in regulating growth and development as well as stress. By analyzing the expression patterns of AhCOL gene family in different tissues, we can understand their potential biological functions. The results of expression patterns in different tissues are shown in most AhCOL genes were expressed in leaves, flowers, and fruit needles. Only two genes, *AhCOL9* and *AhCOL18*, were highly expressed in roots. *AhCOL5*, *AhCOL6*, *AhCOL9*, and *AhCOL15* were abundantly expressed during ExpPod development. Therefore, these genes are widely involved in the growth and development of peanuts. In addition, it was reported that the expression of In Andrographis paniculata and *ApCOL08* was significantly induced by hormone and salt stress. *ApCOL08* was transformed into yeast and showed significant resistance to salt stress [24]. Col transcription factors have the function of stress response in many species. In peanuts, members of the AhCOL gene family may play a crucial role in abiotic stress. Most of the AhCOL members in peanuts contain a large number of ABA and MeJA elements. Additionally, some members have low-temperature response elements, anaerobic induction, circadian control, and defense stress elements. *AhCOL4*, *AhCOL8*, *AhCOL13*, *AhCOL14*, and other genes were induced in the absence of mechanical pressure, with a more pronounced expression under light induction. In this study, the related genes involved in abiotic stress were excavated. It provides an important theoretical basis for the subsequent study of the function of these genes in peanuts.

The functions of CO homologs vary among species. For example, in *Arabidopsis*, the overexpression of *AtCO*, *AtCOL5*, and *AtCOL16* promotes flowering under both long-day (LD) and short-day (SD) conditions [25,26], whereas *AtCOL7*, *AtCOL8*, and *AtCOL9* inhibit flowering under LD conditions [27,28]. In *Oryza sativa*, the *OsHd1* gene delays flowering under LD but promotes it under SD [29], while *OsCOL16* inhibits flowering under both conditions [30]. The *Solanum tuberosum* homolog *StCO* regulates flowering [31]. In Fuji apple, *MdCOL1* and *MdCOL2*, homologs of CO, are critical for reproductive organ development [32]. The *CO* gene, conserved across multiple plant species, is a key component in this regulatory network [18,21,33,34]. AhCOL gene family members were highly expressed in flowers, leaves and Aerpeg. Therefore, the members of AhCOL gene family play a key role in the process of flower development. COP1 (CONSTITUTIVELY PHOTOMORPHOGENIC 1), an E3 ubiquitin ligase in the cryptochrome signaling pathway, binds to and degrades CO via its WD-repeat domain. Members of the *Arabidopsis thaliana* SPA (SUPPRESSOR OF PHYA 105) family also contain a WD domain similar to COP1 and indirectly degrade CO [35]. In Y2H experiments, AhCOL proteins interacted with AhCOP1 and AhNF-YC family members, AhCOL1 interacts with AhNF-YC1, while AhCOL3 interacts with both AhNF-YC1 and AhCOP1 proteins. These two genes have similar expression patterns, and their expression levels are higher in leaves and flowers. These results suggest that the two genes play a key role in the regulation of peanut flowering. This study identified candidate AhCOL genes involved in peanut pod development, providing a theoretical foundation for future functional studies. The CONSTANS-like (COL) gene family plays critical roles in plant growth, development, stress responses, and light signal transduction. CO and its homologous genes have complex regulatory mechanisms in the regulation of plant flowering. So far, there are limited studies on the role of AhCOL genes in peanut development. At present, there is no comprehensive or systematic analysis of AhCOLs family in peanuts. This greatly limits the study of AhCOLs in peanuts. In this study, AhCOLs in cultivated peanuts were identified, and their subcellular locations, gene structure, cis-elements, evolutionary relationships, transcriptional expression, and interaction networks were analyzed to give a comprehensive understanding of AhCOLs in peanuts. Furthermore, the expression patterns of these genes in different tissues and under light and mechanical stress were analyzed. This study provides a theoretical basis for further study on the function of AhCOLs in peanut flowering and abiotic stress.

## 4. Materials and Methods

### 4.1. Experimental Materials

The whole-genome data, including DNA, CDS, protein sequence, and genome annotation files of the *Arabidopsis thaliana*, were obtained from the TAIR database (https://www.arabidopsis.org (accessed on 5 November 2024)). Genome data for *Arachis hypogaea* cv. Tifrunner, a runner-type peanut (registration number CV-93, PI 644011), were sourced from PeanutBase (https://www.peanutbase.org (accessed on 5 November 2024)). Peanuts planted in the greenhouse of the Institute of Advanced Agricultural Sciences of Peking University for 40 days were used as materials.

### 4.2. Identification of AhCOL Gene Family Members in Arachis hypogaea

The conserved domains of the B-box and CCT families were retrieved from the Pfam database (https://www.ebi.ac.uk/interpro/entry/pfam/PF04146/hmm (accessed on 11 November 2024)). *AhCOL* gene family members were identified by aligning these domains to the peanut reference genome using HMMER software (E-value < 1 × 10^−5^). Protein sequences were submitted to the CDD database (https://www.ncbi.nlm.nih.gov/Structure/cdd/wrpsb.cgi (accessed on 11 November 2024)) for confirmation of conserved domains.

### 4.3. Molecular Characterization and Chromosomal Location of AhCOL Genes

Genomic positional data for *AhCOL* genes were extracted from the peanut genome GFF file and visualized using TBTools. Key protein characteristics, including amino acid length, molecular weight, and isoelectric point, were predicted using the ExPASy ProtParam tool (https://web.expasy.org/protparam/ (accessed on 20 November 2024)). Subcellular localization predictions were performed with Plant-mPLoc (http://www.csbio.sjtu.edu.cn/bioinf/plant-multi/#Plant-MPLOC (accessed on 21 November 2024)) [36,37,38,39,40].

### 4.4. Phylogenetic and Genetic Structure Analysis of AhCOL Genes

Phylogenetic trees of AhCOL protein sequences from *Arabidopsis thaliana* and *A. hypogaea* were constructed using MEGA11 software. Phylogenetic trees were generated by the maximum likelihood (ML) method with a bootstrap value of 1000 [41,42]. Motif analysis was conducted using MEME Suite(https://meme-suite.org/meme/tools/meme (version: 5.5.7 accessed on 30 November 2024)), with the motif number set to 10 and the following parameters: the width of the motif ranged from 6 to 50 amino acids. Other options used the default values [43]. Gene cluster analysis, gene structure, and conserved domain were visualized with TBtools (version:2.154) [44].

### 4.5. Predictive Analysis of Promoter Elements of AhCOL Gene in Peanut

Promoter sequences (2 kb upstream of AhCOL genes) were extracted using TBTools and analyzed for *cis*-regulatory elements in the PlantCARE database (https://bioinformatics.psb.ugent.be/webtools/plantcare/html/ (accessed on 30 November 2024)). Synteny analyses were performed using TBtools (version:2.154) (Quick MCScanX Wrapper program with default parameters. The soybean (*Glycine max*) genome data from SoyBase (https://data.soybase.org/Glycine/max (accessed on 30 November 2024)). Promoter elements and synteny results were visualized with TBtools (version:2.154) [44].

### 4.6. RNA-Seq and Bioinformatics Analysis

Previously published transcriptome datasets from Clevenger et al. [19]. were used to profile AhCOL gene expression across peanut tissues. Cui et al.’s transcriptomic data [45] were employed to examine gene expression under light and mechanical stress. A total of 3 pods [46] were retrieved 10–15 days after penetration into the soil and were named DM. The pods were immediately wrapped in a breathable black paper bag and named Sample D (black). Peanut pods were exposed to air are named sample L (light). The D (dark and lose mechanical stress) samples harvested after 10 (day 1), 34 (day 2), and 58 (day 3) h of treatment were named D1, D2, and D3, respectively. The L (light and lose mechanical stress) samples were harvested after 10 (day 1), 34 (day 2), and 58 (day 3) h of treatment and were named L1, L2, and L3. The pod shells of the sample DM, D1, D2, D3, L1, L2, L3, and the seed of sample D3 (named D3S) and L3 (named L3S). Each sample contained two biological replicates.

### 4.7. Subcellular Localization

Tobacco plants were grown under 14 h light/10 h dark cycles at 25 °C and 70% humidity for 4–5 weeks. A subcellular localization vector pFAST-R05-AhCOL-GFP containing the coding region of the target gene was then constructed by the gateway LR reaction and transformed into *E. coli* DH5α and confirmed by PCR and sequencing. The empty vector plasmidpFAST-R05-GFP and the obtained positive recombination subcellular localization vector plasmid pFAST-R05-AhCOL-GFP were transformed into *Agrobacterium tumefaciens* GV3101. Expression plasmids were transformed into *Agrobacterium tumefaciens*, The *Agrobacterium* cells were added to 40 mL YEP liquid culture and oscillation at 28 °C and 200 rpm for 24 h. The collected *A. tumefaciens* (OD_600_ = 0.8–1.0) added into infiltration buffer (10 mM MgCl_2_, 10 mM MES, 150 μM acetosyringone, pH 5.6). The bacterial suspension (OD_600_ = 1.0) was infiltrated into the abaxial surface of *Nicotiana* leaves. After 48 h in darkness, the epidermis was imaged via confocal microscopy to assess fluorescence. Subcellular localization was observed using a fluorescence confocal microscope (Leica, Wetzlar, Germany).

### 4.8. Yeast Two-Hybrid Analysis

Yeast two-hybrid (Y2H) interactions were evaluated using the Y2HGold-GAL4 system (Coolaber, Beijing, China). Positive (Y2HGold [pGBKT7-53 + pGADT7-T]) and negative (Y2HGold [pGBKT7-Lam + pGADT7-T]) controls were included. Coding sequences of AhCOL and AhCOP were cloned into pGADT7 and pGBKT7 vectors, respectively, and transformed into *Saccharomyces cerevisiae* strain AH109. Transformants were cultured on -Leu/-Trp medium and later transferred to -Leu/-Trp/-His/-Ade medium with X-gal for interaction verification. Primers used for amplifying these fragments for yeast two-hybrid assays are listed in Supplementary Table S1.

## Figures and Tables

**Table 1:** Physicochemical properties of members of AhCOL gene family in peanut.

AhCOL Member	Gene ID (arahy.Tifrunner. gnm2.ann1.)	Chromosome Location	Strand	Number of Amino Acids	Molecular Weight/Da	PI	Subcellular Location
*AhCOL1*	arahy.6J3MKV.1	Chr01: 96,565,988_96,569,275	+	364	39,606.40	6.59	Nucleus.
*AhCOL2*	arahy.8MV22J.1	Chr03: 36,453,808_36,456,469	+	434	48,577.11	5.42	Nucleus.
*AhCOL3*	arahy.S3IANQ.1	Chr04: 125,410,273_125,412,732	−	402	43,764.84	5.41	Nucleus.
*AhCOL4*	arahy.7A5VUD.1	Chr06: 8,891,338_8,893,972	+	344	38,530.81	6.17	Nucleus.
*AhCOL5*	arahy.P8XRWE.1	Chr06: 100,018,431_100,023,293	+	432	47,768.07	5.94	Nucleus.
*AhCOL6*	arahy.NK4UTA.1	Chr07: 26,461,465_26,467,006	+	416	45,150.21	5.14	Nucleus.
*AhCOL7*	arahy.WVH2H8.1	Chr09: 113,590,832_113,592,875	−	423	47,372.99	6.24	Nucleus.
*AhCOL8*	arahy.ZS9VWH.1	Chr10: 96,097,643_96,100,039	−	327	35,690.76	6.51	Nucleus.
*AhCOL9*	arahy.LV381L.1	Chr10: 112,354,889_112,357,717	+	351	38,906.28	5.34	Nucleus.
*AhCOL10*	arahy.GMWG2V.1	Chr11: 147,626,175_147,629,467	−	363	39,479.34	6.96	Nucleus.
*AhCOL11*	arahy.ZUL46H.1	Chr13: 38,639,582_38,642,247	+	434	48,523.97	5.48	Nucleus.
*AhCOL12*	arahy.669CET.1	Chr14: 139,785,864_139,788,159	−	403	44,104.19	5.51	Nucleus.
*AhCOL13*	arahy.WEZ503.1	Chr15: 145,833,862_145,836,316	−	404	46,144.44	5.48	Nucleus.
*AhCOL14*	arahy.N2A79V.1	Chr16: 10,817,157_10,819,819	−	344	38,510.83	6.17	Nucleus.
*AhCOL15*	arahy.TFJ3CW.1	Chr17: 30,349,549_30,355,089	−	415	45,053.10	5.14	Nucleus.
*AhCOL16*	arahy.AY8I6Y.1	Chr19: 155,464,256_155,466,032	+	432	48,501.95	6.04	Nucleus.
*AhCOL17*	arahy.B9QMIW.1	Chr20: 118,304,787_118,306,945	−	327	35,721.79	6.51	Nucleus.
*AhCOL18*	arahy.E88P3Q.1	Chr20: 138,909,248_138,911,716	+	392	43,383.72	4.82	Nucleus.

## Data Availability

The RNA-Seq data used in this study are derived from NCBI (http://www.ncbi.nlm.nih.gov/ (accessed on 21 November 2024)) with the submission accession number, SRA: PRJNA291488 and PRJNA770556.

## Supplementary Materials

The supporting information can be downloaded at: https://www.mdpi.com/article/10.3390/ijms26073404/s1.
