# Supplementary material for: Genome-Wide Analysis and Expression Profiles of AhCOLs Family in Peanut (Arachis hypogaea L.)
Source: Int J Mol Sci. 2025 Apr 5;26(7):3404. doi: 10.3390/ijms26073404 (PMC11989928; doi:10.3390/ijms26073404)
Supplement: Supplementary file 1 [file ijms-26-03404-s001.zip › ijms-3495972-supplementary table.pdf]

Table S1 Primer sequences for yeast two-hybrid Synthesized by Sangon Biotech

Primer name	Sequence
AhNF-YC1-BD-F	5′-aggacctgcatatggccatggATGGAGAACACCACCAACACCA-3′
AhNF-YC1-BD-R	5′-tcgacggatccccgggaattcTTATATATGAAAACACCTCTGTCCATCA-3′
AhCOP1-BD-F	5′-aggacctgcatatggccatggATGGAGGACTTCTCTGCTGGG-3′
AhCOP1-BD-R	5′-tcgacggatccccgggaattcTCAAGCTGCAAGCACCAATACT-3′
AhCOL3-AD-F	5′-gccatggaggccagtgaattcATGTTGGACGAAGATACCAA-3′
AhCOL3-AD-R	5′-cagctcgagctcgatggatccGAATGAAGGAACAATGCCAT-3′
AhCOL1-AD-F	5′-gccatggaggccagtgaattcATGGGCCTTAAGGGGTTGA-3′
AhCOL1-AD-R	5′-cagctcgagctcgatggatccAAACGATGGTACGACGCCGT-3′
