# Supplementary material for: Genome-Wide Analysis and Expression Profiles of AhCOLs Family in Peanut (Arachis hypogaea L.)
Source: Int J Mol Sci. 2025 Apr 5;26(7):3404. doi: 10.3390/ijms26073404 (PMC11989928; doi:10.3390/ijms26073404)
Supplement: Supplementary file 1 [file ijms-26-03404-s001.zip › Supplemental Figure.pdf]

Genome-Wide Analysis and Expression Profiles of AhCOLs family in Peanut (*Arachis hypogaea* L.)

Wei Wang[1,2], Xiaoyu Liu[1,2], Che Liu[1], Xiaoqin Liu[1,2*]

1 Peking University Institute of Advanced Agricultural Sciences, Shandong Laboratory of Advanced Agri-culture Sciences at Weifang, Weifang, Shandong, 261325, China.

2 College of Life Sciences, Shandong Agricultural University, Taian, Shandong, 271018, China.

* Correspondence: Xiaoqin Liu; Email: xiaoqin.liu@pku-iaas.edu.cn

Supplementary figure

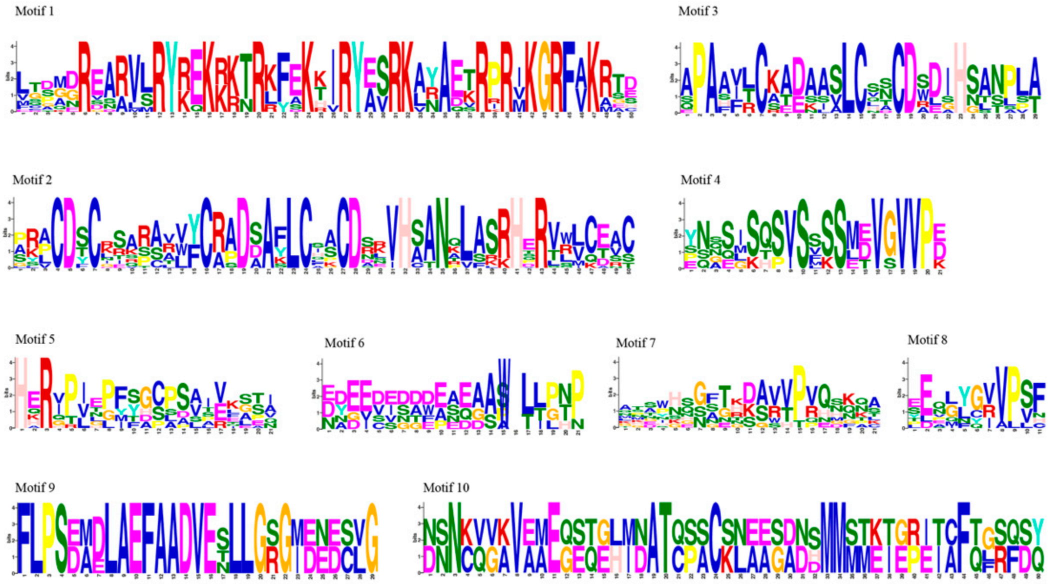

Figure S1 The top ten motif sequence logos of AhCOL gene family, the abscissa represents the amino acid with the highest frequency, and the ordinate represents the relative frequency of the corresponding amino acid.

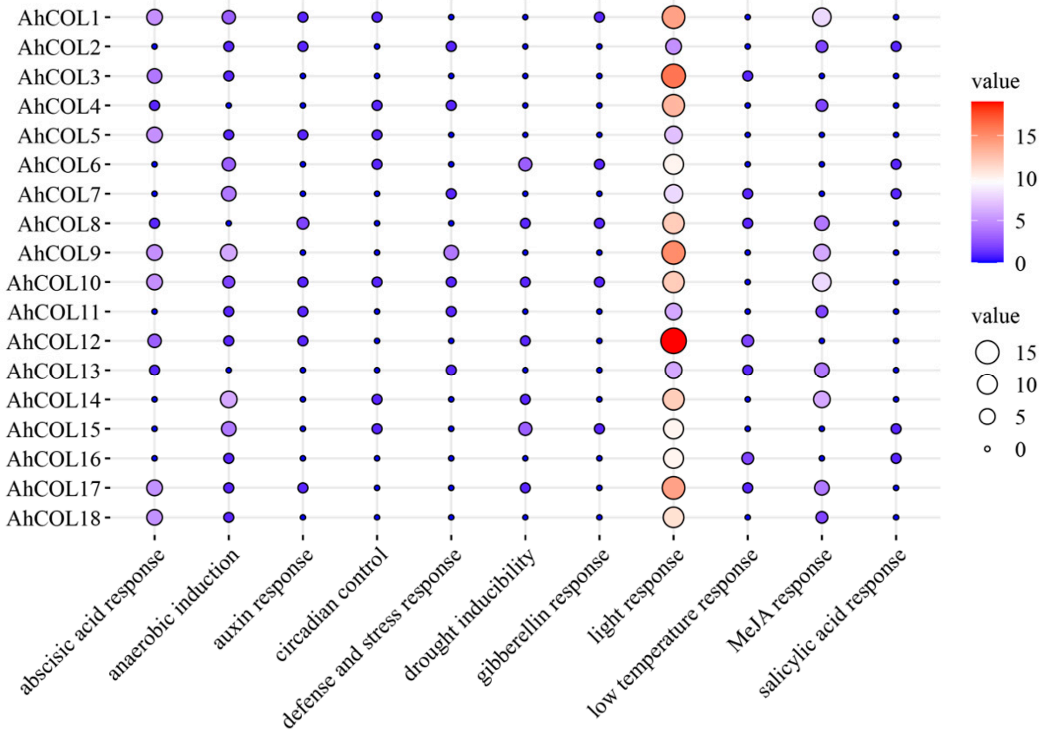

Figure S2 Statistics of the number of elements on the promoter of AhCOL

gene in peanut.

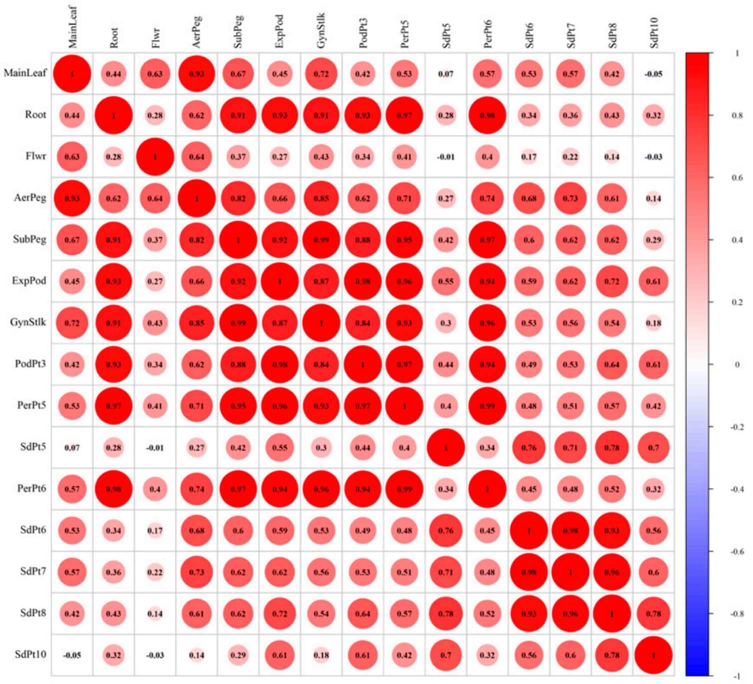

Figure S3 Correlation analysis of AhCOL gene expression in different tissues of peanut.

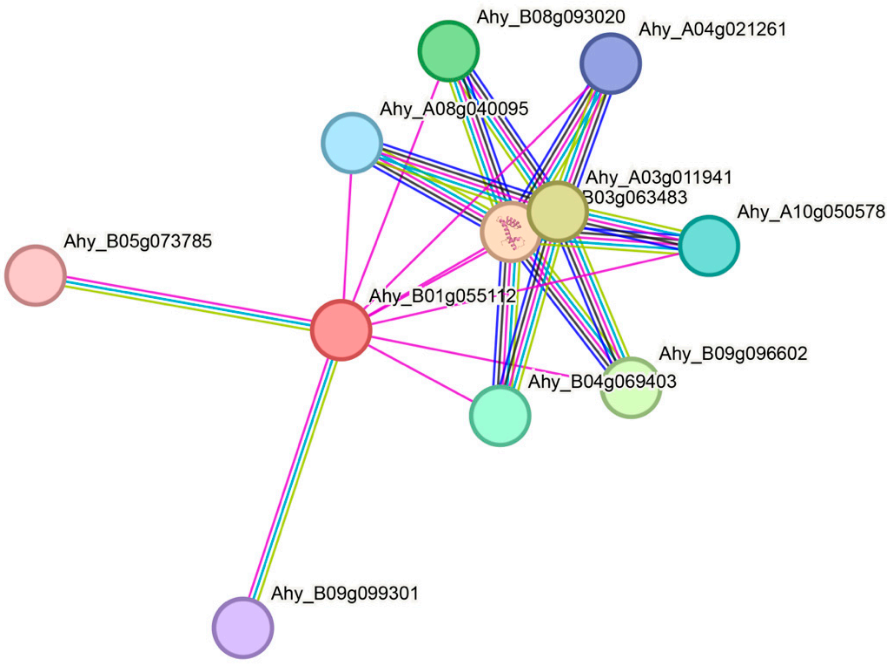

Figure S4 Analysis of AhCOL1 protein network in peanut.

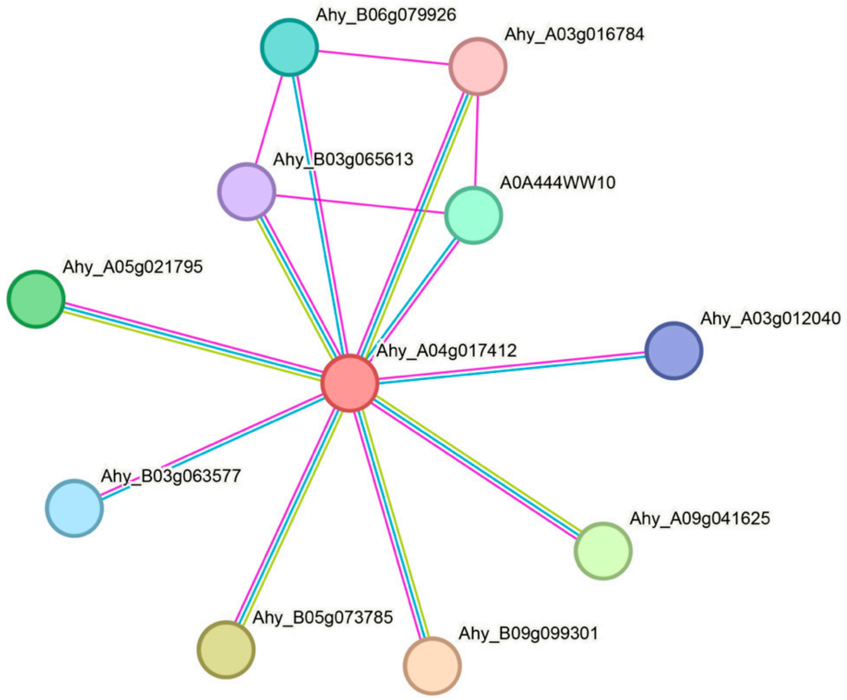

Figure S5 Analysis of AhCOL3 protein network in peanut.
